# Potential interactions between creatine supplementation and prescription drugs: a narrative review and evidence-mapping perspective

**DOI:** 10.1016/j.curtheres.2026.100844

**Published:** 2026-09-06

**Authors:** Sergej M. Ostojic

**Affiliations:** Department of Nutritional Sciences, Texas Tech University, Lubbock, TX, United States

**Keywords:** Creatine, Creatine-drug interactions, Renal excretion, Polypharmacy, Bioenergetics

## Abstract

**Background:**

Creatine is one of the most widely used dietary supplements worldwide and is increasingly consumed by non-athletic populations, including older adults and individuals with chronic metabolic, neurological, and musculoskeletal conditions. Given the high prevalence of prescription drug use in these populations, concomitant use of creatine and medications is common. Several biological pathways suggest potential creatine-drug interactions, particularly related to renal handling and biomarker interpretation; however, the clinical relevance of such interactions remains unclear.

**Methods:**

A PRISMA-aligned narrative and evidence-mapping review was conducted. PubMed (MEDLINE), Scopus, and the Cochrane Library (CENTRAL) were searched without date restrictions for human studies evaluating oral creatine supplementation in the context of concomitant prescription drug use, pharmacokinetics, or interaction-related safety outcomes. Title and abstract screening was performed using predefined eligibility criteria. In parallel, major drug-drug interaction databases (UpToDate, DDInter, Medscape, DrugBank, Drugs.com, the Merck Manual, and the FDA Drug Development and Drug Interactions resource) were queried to contextualize potential interaction signals.

**Results:**

Database searches identified 270 records, of which 132 unique articles remained after deduplication; none met inclusion criteria for full-text evaluation as human studies directly assessing creatine-drug interactions. Across drug-drug interaction databases, no clinically established creatine-drug interactions of moderate or high severity were identified. Reported interaction signals were uniformly classified as minor or theoretical and were primarily attributed to renal excretion–based mechanisms, without evidence of confirmed adverse clinical outcomes.

**Conclusions:**

Despite widespread real-world co-use of creatine supplements and prescription medications, no controlled human studies have directly evaluated creatine-drug interactions. Available evidence from creatine-drug interaction resources suggests predominantly low-severity, theoretical interaction signals rather than clinically confirmed interaction risk. However, the absence of documented clinical evidence should not be interpreted as evidence that interactions cannot occur, particularly in high-risk populations. These findings highlight a substantial evidence gap and underscore the need for targeted human studies to inform clinical guidance.

## Introduction

Creatine is among the most widely consumed dietary supplements worldwide.^1^ Although initially popularized for its ergogenic effects in sport, creatine supplementation is now used across the lifespan and extends well beyond athletic populations.^2^ Its use has expanded in recent years to include older adults and individuals with chronic metabolic, neurological, and musculoskeletal conditions.^3^ Creatine supplements are inexpensive, readily available without prescription, and often consumed continuously over prolonged periods.^4^

Population-based data indicate that creatine use is not confined to sport or exercise settings. In a large U.S. survey, approximately 3% of adults reported using creatine supplements,^5^ with substantially higher prevalence (up to 40%) observed in selected subgroups.^6^ More recently, Benton and co-workers^7^ reported creatine use in 46% of young adults, 32% of middle-aged adults, and 6% of older adults, with no significant differences in dose or frequency across age groups. Collectively, these findings indicate that creatine supplementation is common in the general population and frequently overlaps with prescription drug therapy, particularly among older adults who are more likely to manage chronic conditions and engage in polypharmacy.^8^

From a biological standpoint, several pathways exist through which creatine supplementation and pharmacotherapy could plausibly interact. Oral creatine increases intracellular creatine and phosphocreatine stores, influences cellular energy buffering and high-energy phosphate metabolism, and enhances creatine turnover and renal excretion.^9^ Exogenous creatine may also affect circulating creatinine concentrations, a widely used biomarker for estimating renal function, thereby complicating interpretation of renal indices that are commonly employed to guide drug dosing and monitor adverse effects.^10^ In addition, the osmotic properties of creatine and its potential effects on cellular hydration and renal handling^11^ can intersect with mechanisms relevant to diuretics, nephroactive agents, and drugs that influence systemic or renal hemodynamics. Conversely, commonly prescribed medications, including antihypertensives,^12^ lipid-lowering therapies^13^, and antidiabetic agents,^14^ can modify physiological systems that are relevant to cellular energetics and creatine disposition. However, these mechanisms are largely theoretical and have not been empirically demonstrated in controlled human studies.

Despite these plausible points of intersection, the clinical relevance of potential creatine-drug interactions remains poorly characterized. Dietary supplements such as creatine are not routinely evaluated for interaction potential during product development or post-market surveillance,^15^ and clinical trials of both supplements and pharmaceuticals seldom document concomitant supplement use in a systematic manner.^16^ Consequently, the creatine literature is dominated by studies of efficacy and general safety, with little attention paid to co-use with prescription medications. This gap is particularly notable given the high prevalence of polypharmacy in aging and chronically ill populations,^17^ which increasingly overlap with demographics targeted for creatine supplementation. Although drug-drug interaction databases and prescribing resources generally report no established interactions involving creatine, such findings more likely reflect limited investigation rather than definitive evidence of non-interaction. The present article addresses this gap by critically examining the available evidence on creatine supplementation and concomitant medication use. Rather than drawing definitive conclusions from a sparse literature, we aim to contextualize existing findings in light of biological plausibility, interaction database signals, and real-world patterns of creatine-drug co-use, and to outline priorities for future research in this underexplored area.

## Methods

### Protocol and reporting framework

This review was conducted in accordance with the Preferred Reporting Items for Systematic Reviews and Meta-Analyses (PRISMA) 2020 statement,^18^ adapted to a narrative review with an evidence-mapping approach. The review adopted a PRISMA-aligned methodology for literature identification, study selection, and reporting, while its primary objective was to systematically identify and characterize the available human evidence regarding potential interactions between creatine supplementation and concomitant prescription medication use rather than to quantitatively synthesize treatment effects. The evidence-mapping component comprised a structured evaluation of major clinical drug interaction resources to identify documented creatine–drug interaction signals, summarize their reported severity, proposed mechanisms, and clinical implications, and compare findings across resources.

Given the anticipated scarcity of eligible studies and the exploratory nature of the research question, the review was not prospectively registered. Although prospective registration is recognized as good practice for enhancing transparency and minimizing reporting bias, methodological rigor was maintained through predefined eligibility criteria, a reproducible search strategy, standardized study selection procedures, and transparent reporting in accordance with PRISMA 2020. Future updates of this review, particularly if sufficient primary evidence becomes available to support a formal systematic review, will be prospectively registered in an appropriate registry. A completed PRISMA 2020 checklist is provided as Supplementary Table S1, and the study selection process is summarized in a PRISMA 2020 flow diagram (Supplementary Figure S1).

### Literature search strategy

A targeted literature search was conducted across three electronic databases: PubMed (MEDLINE), Scopus, and the Cochrane Library (CENTRAL), to identify studies examining potential interactions between oral creatine supplementation and concomitant prescription drug use. These databases provide complementary coverage of biomedical, clinical, and pharmacological research and are widely recommended for evidence syntheses in health sciences. The search strategy was intentionally designed to maximize specificity for supplement-drug interaction content and to minimize retrieval of studies focused exclusively on exercise performance, sports nutrition, or basic creatine biology. In each database, searches were restricted to title and abstract fields (or their database-specific equivalents) and used combinations of terms related to creatine supplementation and drug interaction concepts. Core search terms included “creatine monohydrate” and “creatine supplementation”, combined with keywords denoting interaction or pharmacological relevance, such as “drug interaction,” “concomitant medication”, “pharmacokinetic”, and related truncations. Database-specific syntax and field tags were adapted as appropriate. No restrictions were applied with respect to publication date. Searches were limited to articles published in English and involving human subjects. Reference lists of relevant articles were screened manually to identify additional records that may not have been captured through electronic searches.

### Eligibility criteria

Studies were considered eligible for inclusion if they met all of the following criteria: (1) involved oral creatine supplementation as an exposure of interest; (2) included concomitant prescription medication use, pharmacokinetic assessment, or interaction-related safety outcomes; (3) were conducted in human participants; and (4) reported original data. For the purposes of this review, prescription medications were broadly defined as medications requiring a prescription for clinical use, irrespective of therapeutic indication or pharmacological class. No restrictions were placed on therapeutic class, and any human study evaluating creatine supplementation in the context of concomitant prescription medication use was considered eligible. This included, but was not limited to, cardiovascular, antihypertensive, antidiabetic, lipid-lowering, anti-inflammatory, immunosuppressive, anti-infective, neurological, psychiatric, and oncological medications. Studies were excluded if they were conducted exclusively in animal or in vitro models; were review articles, editorials, commentaries, or guidelines without original data; focused solely on exercise performance, training adaptations, or sports outcomes without consideration of medication use; or mentioned creatine or prescription medications incidentally without evaluating interaction-related outcomes.

### Study selection and data handling

All records retrieved from the three databases were pooled, and duplicate entries were removed based on normalized article titles. Title and abstract screening was conducted by a single reviewer (SMO) using predefined eligibility criteria and a standardized screening procedure. Although duplicate independent screening is generally recommended to reduce the risk of selection bias, this approach was not feasible because of resource constraints. Given the absence of eligible records after initial screening, no full-text assessments were conducted. For records meeting preliminary relevance thresholds, data extraction was planned to include study design, participant characteristics, creatine formulation and dose, drug(s) involved, interaction-related outcomes, and reported safety findings. No formal risk-of-bias or study quality assessment was undertaken because no studies met the predefined eligibility criteria and therefore none progressed to full-text review. Consistent with the objectives of this PRISMA-aligned evidence-mapping review, methodological transparency was instead supported through predefined eligibility criteria, a reproducible search strategy, and comprehensive reporting in accordance with PRISMA 2020, including the accompanying checklist and flow diagram.

### Data synthesis

Owing to the absence of eligible studies and the heterogeneity of excluded literature, findings were synthesized narratively. Quantitative synthesis or meta-analysis was not feasible. The narrative synthesis focused on identifying gaps in the literature and contextualizing findings using complementary sources of evidence.

### Use of drug-drug interaction databases

To contextualize the absence of primary clinical evidence, several major drug-drug interaction databases were queried for reported interactions involving creatine. These included UpToDate (https://www.uptodate.com), DDInter (https://ddinter.scbdd.com/), Medscape Drug Interaction Checker (https://reference.medscape.com/drug-interactionchecker), DrugBank (https://go.drugbank.com/), Drugs.com (https://www.drugs.com/), the Merck Manual Professional Edition (https://www.merckmanuals.com/professional), and the FDA Drug Development and Drug Interactions resource (https://www.fda.gov/drugs/drug-interactions-labeling/drug-development-and-drug-interactions-resources). These databases were consulted to identify any documented evidence of interactions involving creatine. These resources were selected because they represent major academic, regulatory, and clinician-oriented references commonly consulted for drug interaction information. Each database was searched independently using the terms creatine, creatine monohydrate, and related entries where supported by the platform. Searches were conducted using a standardized approach across all resources, and the presence or absence of documented creatine–drug interactions was recorded. This supplementary evidence-mapping exercise was intended to complement, rather than replace, the systematic literature search.

## Results

Searches of Scopus, PubMed, and Cochrane CENTRAL identified 270 records prior to deduplication, including 120 from Scopus, 95 from PubMed, and 55 from Cochrane CENTRAL. Following removal of 138 duplicate records based on normalized article titles, 132 unique articles remained for title and abstract screening. None met the predefined eligibility criteria for human studies evaluating creatine supplementation in the context of concomitant prescription medication use, pharmacokinetic assessment, or interaction-related safety outcomes. Specifically, no clinical trials, observational studies, pharmacokinetic investigations, or case reports evaluating clinically relevant creatine–drug interactions were identified. Excluded articles primarily did not evaluate drug interactions, involved animal or in vitro models, consisted of reviews, editorials, or commentaries without original data, or focused exclusively on exercise performance or training adaptations without consideration of concomitant medication use. Consequently, no reports progressed to full-text assessment or qualitative synthesis. The study selection process is summarized in the PRISMA 2020 flow diagram (Supplementary Figure S1).

### Drug-drug interaction database findings

Queries of major drug-drug interaction databases revealed no clinically established creatine-drug interactions of moderate or high severity (Table 1). UpToDate reported no interactions classified as Risk Level A or higher involving creatine supplementation, and DrugBank did not list any documented creatine-drug interactions. The FDA Drug Development and Drug Interactions resource, which primarily catalogs CYP enzyme- and transporter-mediated interactions relevant to pharmaceutical drug development, likewise did not identify creatine-related interaction signals. Review of the Merck Manual revealed no documented drug interactions with creatine, although precautionary notes were provided regarding concomitant use with medications affecting renal function and with caffeine.

**Table 1:** Summary of creatine–drug interaction findings across major drug-drug interaction databases.

Database	Interactions identified	Highest severity reported	Drugs / Drug classes implicated	Primary mechanism noted	Documented clinical outcomes
UpToDate	None	None	None	Not applicable	None reported
DrugBank	None	None	None	Not applicable	None reported
FDA Drug Development and Drug Interactions	None	None	None	CYP enzyme and transporter-based framework	Not applicable
Merck Manual	None documented	None	NSAIDs (precautionary note), caffeine	Renal function considerations; energy metabolism	None reported
DDInter	5 interactions	Moderate	Pemetrexed, entecavir (moderate) Cimetidine, trimethoprim, probenecid (minor)	Renal excretion / tubular competition	None reported
Medscape	46 interactions	Minor	NSAIDs, salicylates, ACE inhibitors; cyclosporine, entecavir, sulfasalazine	Renal function / hemodynamic effects	None reported
Drugs.com	6 interactions	Monitoring-level	Cimetidine, entecavir, pemetrexed, probenecid, trimethoprim, trospium	Active renal tubular secretion	None reported

Note. Drug interaction resources varied in their classification systems and terminology. Most identified interactions represented theoretical, precautionary, or monitoring-based alerts derived from pharmacokinetic considerations (primarily renal tubular secretion or renal function) rather than clinically confirmed adverse drug–creatine interactions. No controlled human studies demonstrating clinically significant creatine–drug interactions were identified through the accompanying systematic literature review.

Interrogation of the DDInter database identified a limited number of interaction entries involving creatine, all attributed to renal excretion-based mechanisms. Two interactions were classified as moderate (pemetrexed and entecavir), and three as minor (cimetidine, trimethoprim, and probenecid). All entries were based on predicted competition for renal excretion pathways, with no interactions classified as severe and no information provided on confirmed clinical outcomes. According to the Medscape Drug Interaction Checker, creatine supplementation was associated with 46 minor interaction flags, primarily involving nonsteroidal anti-inflammatory drugs (NSAIDs) and salicylates as well as angiotensin-converting enzyme (ACE) inhibitors, with additional minor entries for cyclosporine, entecavir, and sulfasalazine. All listed interactions were categorized as minor and attributed to potential additive effects on renal function or renal hemodynamics. The Drugs.com interaction checker identified a small set of medications reported to interact with creatine, including cimetidine, entecavir, pemetrexed, probenecid, trimethoprim, and trospium. All listed interactions were mechanistically linked to active renal tubular secretion, with no interactions categorized as severe and no confirmed adverse clinical outcomes reported.

## Discussion

The present analysis identified a striking gap in the clinical literature on potential creatine–drug interactions. Despite comprehensive searches across PubMed, Scopus, and Cochrane CENTRAL, together with interrogation of multiple authoritative clinical drug interaction resources, no eligible human studies were identified that directly evaluated creatine supplementation in the context of concomitant prescription medication use, pharmacokinetics, pharmacodynamics, or interaction-related safety outcomes. Specifically, no randomized controlled trials, observational studies, pharmacokinetic investigations, or published case reports describing clinically confirmed adverse creatine–drug interactions met the predefined eligibility criteria. Most excluded publications focused on exercise performance, employed non-human models, or investigated creatine supplementation without consideration of concomitant medication use. Given the widespread use of creatine among populations in whom prescription drug use is common, including older adults and individuals with chronic cardiometabolic, neurological, and musculoskeletal conditions^7^, this finding appears to reflect a lack of targeted investigation rather than selective reporting or database-specific bias.

In the absence of primary clinical evidence, clinical drug interaction resources provide a complementary, albeit indirect, source of information. Across most major platforms—including UpToDate, DrugBank, the FDA Drug Development and Drug Interactions resource, and the Merck Manual—no clinically established creatine–drug interactions of moderate or high severity were identified. Databases reporting interaction signals, including DDInter, Medscape, and Drugs.com, consistently attributed these to theoretical pharmacokinetic mechanisms involving shared renal handling, competition for active tubular secretion, or potential additive effects on renal function. Reported precautionary signals primarily involved NSAIDs and salicylates, ACE inhibitors, cyclosporine, entecavir, sulfasalazine, cimetidine, trimethoprim, probenecid, trospium, and pemetrexed. Importantly, these classifications were based on theoretical pharmacological considerations rather than controlled clinical studies demonstrating clinically significant adverse interactions. Accordingly, the reported interaction signals should be regarded as hypothesis-generating rather than clinically validated creatine–drug interactions. The currently available evidence therefore does not support the routine avoidance of creatine supplementation in individuals receiving these medications. Nevertheless, because no well-designed human studies have directly evaluated these combinations, clinicians should continue to exercise individualized clinical judgment, particularly in older adults with polypharmacy, patients with chronic kidney disease, and individuals receiving medications with nephrotoxic potential or those primarily eliminated through renal tubular secretion. Future pharmacokinetic studies, pharmacovigilance investigations, and adequately powered clinical trials are needed to determine whether these theoretical interaction signals translate into clinically meaningful outcomes in high-risk populations.

An additional consideration is that creatine supplementation has been investigated in numerous clinical populations in which participants were likely receiving concomitant prescription medications, including individuals with neurological disorders, metabolic diseases, cardiovascular conditions, and age-related disorders. Although these studies were not designed to evaluate drug–creatine interactions and generally did not systematically document concomitant medication use or interaction-related outcomes, they have not generated a consistent published signal suggesting clinically important adverse interactions attributable to creatine supplementation. Nevertheless, this observation should be interpreted cautiously, as the absence of reported interactions does not constitute evidence that clinically relevant interactions do not occur, particularly in high-risk populations or among individuals receiving medications with narrow therapeutic indices.

A recurring theme across database findings is the role of renal handling, particularly with drugs known to influence creatinine secretion or renal hemodynamics. Agents such as cimetidine, trimethoprim, and probenecid are recognized inhibitors of tubular creatinine secretion,^19^^,^^20^ and their coadministration with creatine may lead to elevations in serum creatinine that reflect altered biomarker handling rather than true reductions in glomerular filtration. This distinction is clinically important, as misinterpretation of creatinine-based estimates of renal function could lead to unnecessary concern or inappropriate modification of drug therapy in individuals using creatine supplements.^21^

Collectively, the above findings suggest that current concerns regarding creatine-drug interactions are largely precautionary and mechanistic in nature, rather than grounded in direct clinical evidence. At the same time, the lack of systematic human studies represents a critical knowledge gap. Given the high prevalence of both creatine supplementation and polypharmacy, particularly in aging populations, even modest interaction effects could have clinical relevance if they exist. The absence of evidence should therefore not be interpreted as definitive proof of non-interaction, but rather as an indication that this area has not been adequately studied. Future research should thus prioritize controlled human studies that explicitly document supplement use, include standardized assessments of renal function beyond serum creatinine, and evaluate clinically meaningful outcomes in populations most likely to use creatine alongside prescription medications. Such studies would provide a stronger empirical basis for clinical guidance than reliance on theoretical database signals alone.

The findings derived from clinical drug interaction resources should be interpreted cautiously because these resources are designed primarily to support clinical decision-making rather than to provide primary scientific evidence. Their interaction classifications are based on varying combinations of published literature, pharmacological principles, regulatory information, expert curation, and proprietary classification algorithms. Consequently, discrepancies between resources are expected and likely reflect differences in evidence selection, update frequency, interaction severity classification systems, and the weighting of theoretical pharmacokinetic mechanisms. This variability was evident in the present review, where some resources identified no interactions while others reported precautionary signals of differing severity. Accordingly, these database-derived signals should be regarded as hypothesis-generating and complementary to, rather than a substitute for, evidence obtained from well-designed human pharmacokinetic and clinical studies.

From a clinical perspective, the findings of this review should be interpreted as reassuring but not definitive. The current evidence does not support routine avoidance of creatine supplementation in individuals receiving prescription medications, nor does it indicate that creatine should be discontinued solely because concomitant pharmacotherapy is initiated. However, given the absence of studies specifically designed to evaluate creatine–drug interactions, clinicians should continue to obtain a comprehensive history of dietary supplement use and consider individualized risk assessment, particularly in older adults with polypharmacy, patients with chronic kidney disease, and individuals receiving medications with nephrotoxic potential or those primarily eliminated through renal tubular secretion. In such circumstances, consideration of periodic monitoring of renal function and clinical status may be appropriate until higher-quality evidence becomes available.

The strengths of this review include a comprehensive, multi-database search strategy, rigorous deduplication and screening procedures, and triangulation of findings across multiple clinical drug interaction resources. Nevertheless, several limitations warrant consideration. First, although the literature search included PubMed, Scopus, and Cochrane CENTRAL, together with supplementary searches of major clinical drug interaction resources, additional bibliographic databases such as Embase and Web of Science were not searched. Although these databases may have identified additional publications, the absence of eligible studies across multiple complementary sources suggests that this limitation is unlikely to have materially affected the principal conclusions of the review. Second, the clinical drug interaction resources examined are designed primarily to support clinical decision-making rather than to provide primary scientific evidence, and they are not specifically intended to systematically evaluate dietary supplements. Consequently, their outputs may be constrained by incomplete data capture, variable evidence curation, differences in interaction classification systems, update frequency, and database-specific algorithms, all of which may contribute to discrepancies between resources. Third, the absence of eligible human studies precluded qualitative or quantitative synthesis and prevented direct assessment of interaction-related risk or causality. Fourth, relevant unpublished studies, regulatory reports, or pharmacovigilance data may exist but were not accessible through the bibliographic databases and clinical drug interaction resources examined. Fifth, study screening was conducted by a single reviewer without independent duplicate verification. Although predefined eligibility criteria and a standardized screening process were used to minimize selection bias, the possibility of inadvertent study selection errors cannot be completely excluded. Finally, creatine use is often self-reported or incompletely documented in both clinical practice and research settings because dietary supplements are frequently omitted from medication histories. This underdocumentation may further limit the detection of potential creatine–drug interactions, particularly when their effects are subtle or manifest primarily as changes in laboratory biomarkers rather than overt clinical events.

## Conclusion

In summary, despite the widespread real-world co-use of creatine supplements and prescription medications, the current literature contains no controlled human studies directly evaluating creatine–drug interactions. Clinical drug interaction resources identified either no interactions or predominantly low-severity, theoretical interaction signals, primarily related to renal excretion mechanisms, without documented adverse clinical outcomes. These findings highlight an important gap between the widespread use of creatine and the evidence available to inform clinical decision-making, underscoring the need for well-designed pharmacokinetic, pharmacodynamic, and clinical studies evaluating the safety of creatine supplementation in individuals receiving concomitant prescription medications. Importantly, the absence of eligible human studies should be viewed as an important evidence gap rather than evidence that clinically relevant creatine–drug interactions do not occur.

## Statement of ethics

Not applicable.

## Funding sources

None.

## Author contributions

Conceptualization: SMO. Methodology: SMO. Formal analysis: SMO. Investigation: SMO. Data curation: SMO. Writing-original draft preparation: SMO. Writing-review and editing: SMO. The author has read and agreed to the published version of the manuscript.

## Data availability statement

Not applicable.

## AI statement

During the preparation of this manuscript, the author used ChatGPT (OpenAI) for language editing and grammatical refinement. All AI-assisted content was subsequently reviewed, verified, and revised by the authors, who take full responsibility for the accuracy, integrity, and final content of the manuscript.

## Consent for publication

The author gives permission for the Journal to publish this work.

## Declaration of competing interest

SMO serves on scientific advisory boards related to creatine. SMO is an inventor on creatine-related intellectual property and has received research funding on creatine from public agencies and industry partners. SMO co-founded a venture developing creatine-enriched food products.
