# Supplementary material for: Potential interactions between creatine supplementation and prescription drugs: a narrative review and evidence-mapping perspective
Source: Curr Ther Res Clin Exp. 2026 Sep 6;105:100844. doi: 10.1016/j.curtheres.2026.100844 (PMC13584064; doi:10.1016/j.curtheres.2026.100844)
Supplement: Supplementary file 1 [file mmc2.pdf]

PRISMA 2020 flow diagram

Creatine supplementation and prescription drug interactions review

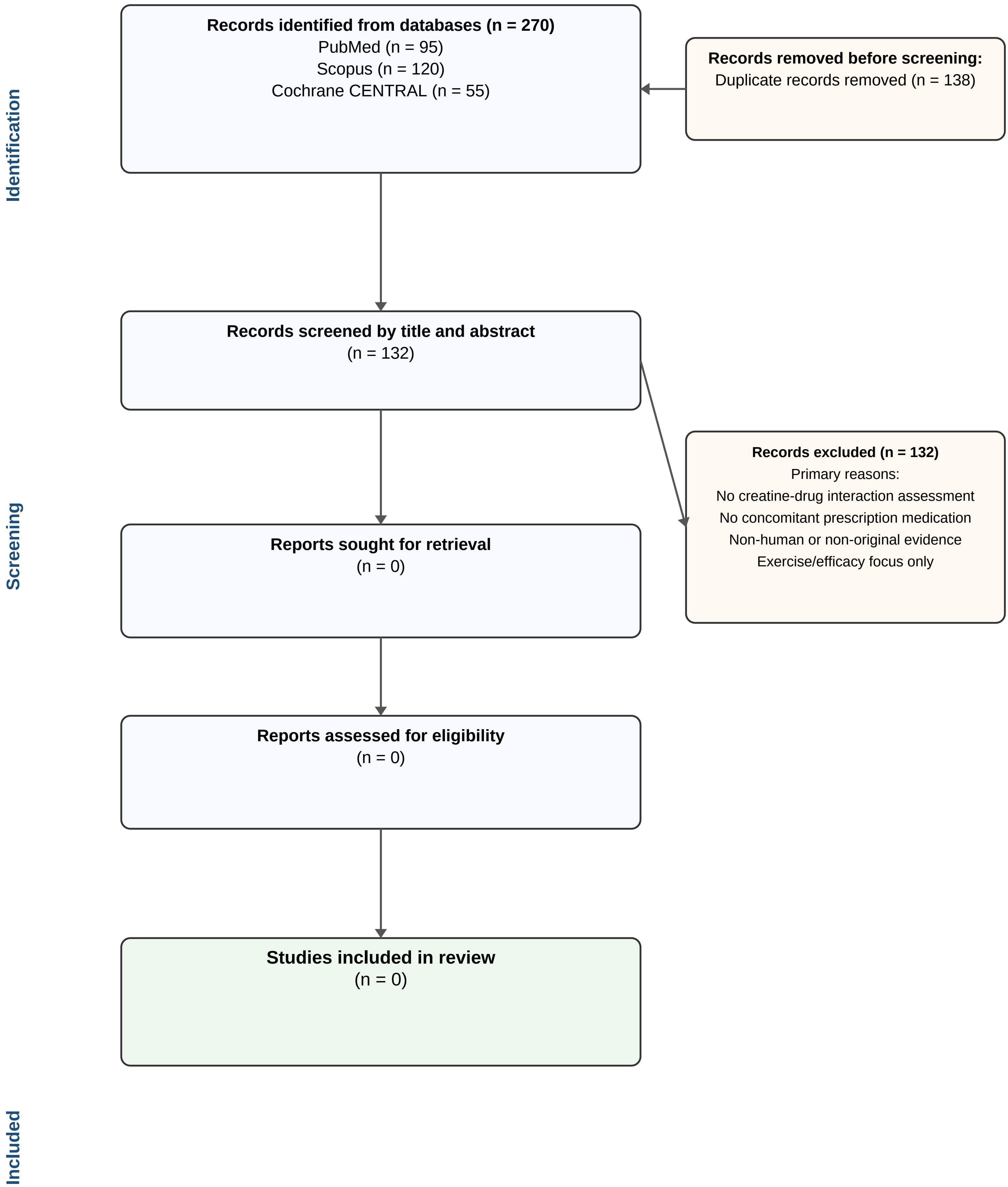

Note: All unique records were excluded at title/abstract screening; therefore, no reports proceeded to full-text retrieval or eligibility assessment.

Prepared in accordance with the PRISMA 2020 study-selection framework.
