# Supplementary material for: Potential interactions between creatine supplementation and prescription drugs: a narrative review and evidence-mapping perspective
Source: Curr Ther Res Clin Exp. 2026 Sep 6;105:100844. doi: 10.1016/j.curtheres.2026.100844 (PMC13584064; doi:10.1016/j.curtheres.2026.100844)
Supplement: Supplementary file 2 [file mmc1.docx]

**PRISMA 2020 Checklist**

**Manuscript: Potential Interactions Between Creatine Supplementation and Prescription Drugs: A Narrative Review and Evidence-Mapping Perspective**

**Completion note:** Locations are given by manuscript section because final page and line numbers were not available. Bracketed entries require completion after the manuscript is revised and paginated. “Not applicable” is used where no eligible studies were identified and the corresponding analyses could not be performed.

| **Section and topic** | **Item #** | **Checklist item** | **Location where item is reported / applicability** |
| --- | --- | --- | --- |
| **TITLE** | **1** | Identify the report as a systematic review. | Title. The report is identified as a narrative review and evidence-mapping review; consider adding “systematic search” or “systematic evidence map” if the journal requires explicit identification as a systematic review. |
| **ABSTRACT** | **2** | See the PRISMA 2020 for Abstracts checklist. | Abstract: Background, Methods, Results, and Conclusions. A separate PRISMA 2020 for Abstracts checklist may also be supplied if requested. |
| **INTRODUCTION** | **3** | Describe the rationale for the review in the context of existing knowledge. | Introduction, paragraphs 1–4. |
| **INTRODUCTION** | **4** | Provide an explicit statement of the objective(s) or question(s) the review addresses. | End of Introduction: the article aims to examine available evidence, contextualize biological plausibility and database signals, and outline research priorities. |
| **METHODS** | **5** | Specify the inclusion and exclusion criteria for the review and how studies were grouped for the syntheses. | Methods—Eligibility criteria. Grouping for synthesis was not applicable because no eligible studies were identified. |
| **METHODS** | **6** | Specify all databases, registers, websites, organisations, reference lists and other sources searched or consulted to identify studies. Specify the date when each source was last searched or consulted. | Methods—Literature search strategy and Use of drug-drug interaction databases. Add exact last-search date for each bibliographic database and interaction resource: [INSERT DATES]. |
| **METHODS** | **7** | Present the full search strategies for all databases, registers and websites, including any filters and limits used. | Not fully reported in the supplied manuscript. Add complete database-specific search strings as Supplementary Table S2, including field tags, filters, and limits. |
| **METHODS** | **8** | Specify the methods used to decide whether a study met the inclusion criteria of the review, including how many reviewers screened each record and each report retrieved, whether they worked independently, and if applicable, details of automation tools used in the process. | Methods—Study selection and data handling: title/abstract screening by one reviewer (SMO) using predefined criteria; no automation tools reported; no full texts were assessed. |
| **METHODS** | **9** | Specify the methods used to collect data from reports, including how many reviewers collected data from each report, whether they worked independently, any processes for obtaining or confirming data from study investigators, and if applicable, details of automation tools used in the process. | Methods—Study selection and data handling. A data-extraction plan is described, but formal extraction was not undertaken because no studies were eligible. No investigator contact or automation tools were used/reported. |
| **METHODS** | **10a** | List and define all outcomes for which data were sought. Specify whether all results that were compatible with each outcome domain in each study were sought, and if not, the methods used to decide which results to collect. | Methods—Eligibility criteria and planned data extraction: interaction-related safety outcomes, pharmacokinetic outcomes, and reported safety findings. No outcome data were collected because no eligible studies were found. |
| **METHODS** | **10b** | List and define all other variables for which data were sought (e.g. participant and intervention characteristics, funding sources). Describe any assumptions made about any missing or unclear information. | Methods—Study selection and data handling: planned variables included study design, participant characteristics, creatine formulation/dose, drugs involved, and interaction-related outcomes. Funding sources and assumptions about missing data were not applicable because no studies were included. |
| **METHODS** | **11** | Specify the methods used to assess risk of bias in the included studies, including details of the tool(s) used, how many reviewers assessed each study and whether they worked independently, and if applicable, details of automation tools used in the process. | Not applicable—no eligible studies were identified; therefore, no risk-of-bias assessment was performed. This is stated in Methods—Study selection and data handling. |
| **METHODS** | **12** | Specify for each outcome the effect measure(s) (e.g. risk ratio, mean difference) used in the synthesis or presentation of results. | Not applicable—no eligible studies and no quantitative synthesis. |
| **METHODS** | **13a** | Describe the processes used to decide which studies were eligible for each synthesis (e.g. tabulating the study intervention characteristics and comparing against the planned groups for each synthesis). | Not applicable—no eligible studies. |
| **METHODS** | **13b** | Describe any methods required to prepare the data for presentation or synthesis, such as handling of missing summary statistics or data conversions. | Not applicable—no data were extracted. |
| **METHODS** | **13c** | Describe any methods used to tabulate or visually display results of individual studies and syntheses. | Methods—Data synthesis and Results—Drug-drug interaction database findings. Database-derived signals were summarized narratively and in Table 1; no individual eligible studies were available. |
| **METHODS** | **13d** | Describe any methods used to synthesize results and provide a rationale for the choice(s). If meta-analysis was performed, describe the model(s), method(s) to identify statistical heterogeneity, and software package(s) used. | Methods—Data synthesis: narrative synthesis was used because no eligible studies were identified; meta-analysis was not feasible. |
| **METHODS** | **13e** | Describe any methods used to explore possible causes of heterogeneity among study results (e.g. subgroup analysis, meta-regression). | Not applicable—no eligible studies or synthesis of study results. |
| **METHODS** | **13f** | Describe any sensitivity analyses conducted to assess robustness of the synthesized results. | Not applicable—no eligible studies or quantitative synthesis. |
| **METHODS** | **14** | Describe any methods used to assess risk of bias due to missing results in a synthesis (arising from reporting biases). | Not applicable—no eligible studies or synthesis. |
| **METHODS** | **15** | Describe any methods used to assess certainty (or confidence) in the body of evidence for an outcome. | Not applicable—no eligible studies and therefore no body of direct clinical evidence to grade. |
| **RESULTS** | **16a** | Describe the results of the search and selection process, from the number of records identified in the search to the number of studies included in the review, ideally using a flow diagram. | Results, first paragraph: 270 records identified; 132 unique records after deduplication; all excluded at title/abstract screening; no full-text evaluations or included studies. Add/confirm PRISMA 2020 flow diagram as Supplementary Figure S1. |
| **RESULTS** | **16b** | Cite studies that might appear to meet the inclusion criteria, but which were excluded, and explain why they were excluded. | No near-eligible full-text reports were identified because all records were excluded at title/abstract screening. If any potentially relevant reports were examined in detail, list them with reasons; otherwise state explicitly that there were no full-text exclusions. |
| **RESULTS** | **17** | Cite each included study and present its characteristics. | Not applicable—no eligible studies were included. |
| **RESULTS** | **18** | Present assessments of risk of bias for each included study. | Not applicable—no eligible studies. |
| **RESULTS** | **19** | For all outcomes, present, for each study: (a) summary statistics for each group (where appropriate) and (b) an effect estimate and its precision (e.g. confidence/credible interval), ideally using structured tables or plots. | Not applicable—no eligible studies. |
| **RESULTS** | **20a** | For each synthesis, briefly summarise the characteristics and risk of bias among contributing studies. | Not applicable—no eligible studies. |
| **RESULTS** | **20b** | Present results of all statistical syntheses conducted. If meta-analysis was done, present for each the summary estimate and its precision and measures of statistical heterogeneity. If comparing groups, describe the direction of the effect. | Not applicable—no statistical synthesis or meta-analysis. |
| **RESULTS** | **20c** | Present results of all investigations of possible causes of heterogeneity among study results. | Not applicable. |
| **RESULTS** | **20d** | Present results of all sensitivity analyses conducted to assess the robustness of the synthesized results. | Not applicable. |
| **RESULTS** | **21** | Present assessments of risk of bias due to missing results (arising from reporting biases) for each synthesis assessed. | Not applicable. |
| **RESULTS** | **22** | Present assessments of certainty (or confidence) in the body of evidence for each outcome assessed. | Not applicable. The Discussion emphasizes the absence of direct human evidence and that absence of evidence should not be interpreted as proof of non-interaction. |
| **DISCUSSION** | **23a** | Provide a general interpretation of the results in the context of other evidence. | Discussion, paragraphs 1–4. |
| **DISCUSSION** | **23b** | Discuss any limitations of the evidence included in the review. | Discussion: absence of direct human studies; database signals are theoretical and not supported by confirmed clinical outcomes. |
| **DISCUSSION** | **23c** | Discuss any limitations of the review processes used. | Discussion—limitations paragraph: limitations of interaction databases, lack of quantitative synthesis, inaccessible unpublished/post-marketing data, and underdocumentation of supplement use. Add prospective non-registration as a limitation if not already added. |
| **DISCUSSION** | **23d** | Discuss implications of the results for practice, policy, and future research. | Discussion and Conclusion: cautious clinical interpretation, need for renal measures beyond serum creatinine, and priority for controlled human studies in populations with polypharmacy. |
| **OTHER INFORMATION** | **24a** | Provide registration information for the review, including register name and registration number, or state that the review was not registered. | Methods—Protocol and reporting framework: review was not prospectively registered. |
| **OTHER INFORMATION** | **24b** | Indicate where the review protocol can be accessed, or state that a protocol was not prepared. | Not explicitly stated. Add: “A separate review protocol was not prepared or published.” |
| **OTHER INFORMATION** | **24c** | Describe and explain any amendments to information provided at registration or in the protocol. | Not applicable—the review was not registered and no separate protocol was prepared. |
| **OTHER INFORMATION** | **25** | Describe sources of financial or non-financial support for the review, and the role of the funders or sponsors in the review. | [INSERT manuscript Funding statement and location]. If none: “This review received no specific funding.” |
| **OTHER INFORMATION** | **26** | Declare any competing interests of review authors. | [INSERT manuscript Conflict of Interest statement and location]. |
| **OTHER INFORMATION** | **27** | Report which of the following are publicly available and where they can be found: template data collection forms; data extracted from included studies; data used for all analyses; analytic code; any other materials used in the review. | No study-level data or analytic code were generated because no eligible studies were included. Add a Data Availability statement and provide full search strategies and completed checklist in the Supplementary Materials. |

**Citation:** Page MJ, McKenzie JE, Bossuyt PM, et al. The PRISMA 2020 statement: an updated guideline for reporting systematic reviews. BMJ. 2021;372:n71. doi:10.1136/bmj.n71.

Checklist adapted from the official PRISMA 2020 checklist, distributed under the Creative Commons Attribution 4.0 International License (CC BY 4.0).
